# Metabolome Integrated Analysis of High-Temperature Response in *Pinus radiata*

**DOI:** 10.3389/fpls.2018.00485

**Published:** 2018-04-17

**Authors:** Mónica Escandón, Mónica Meijón, Luis Valledor, Jesús Pascual, Gloria Pinto, María Jesús Cañal

**Affiliations:** ^1^Plant Physiology, Department of Organisms and Systems Biology, Faculty of Biology, University of Oviedo, Oviedo, Spain; ^2^Plant Biotechnology Unit, University Institute of Biotechnology of Asturias (IUBA), Oviedo, Spain; ^3^Molecular Plant Biology, Department of Biochemistry, University of Turku, Turku, Finland; ^4^Department of Biology and CESAM, University of Aveiro, Aveiro, Portugal

**Keywords:** pine, heat-acclimation, metabolomics, multivariate integrative analyses, biomarkers

## Abstract

The integrative omics approach is crucial to identify the molecular mechanisms underlying high-temperature response in non-model species. Based on future scenarios of heat increase, *Pinus radiata* plants were exposed to a temperature of 40°C for a period of 5 days, including recovered plants (30 days after last exposure to 40°C) in the analysis. The analysis of the metabolome using complementary mass spectrometry techniques (GC-MS and LC-Orbitrap-MS) allowed the reliable quantification of 2,287 metabolites. The analysis of identified metabolites and highlighter metabolic pathways across heat time exposure reveal the dynamism of the metabolome in relation to high-temperature response in *P. radiata*, identifying the existence of a turning point (on day 3) at which *P. radiata* plants changed from an initial stress response program (shorter-term response) to an acclimation one (longer-term response). Furthermore, the integration of metabolome and physiological measurements, which cover from the photosynthetic state to hormonal profile, suggests a complex metabolic pathway interaction network related to heat-stress response. Cytokinins (CKs), fatty acid metabolism and flavonoid and terpenoid biosynthesis were revealed as the most important pathways involved in heat-stress response in *P. radiata*, with zeatin riboside (ZR) and isopentenyl adenosine (iPA) as the key hormones coordinating these multiple and complex interactions. On the other hand, the integrative approach allowed elucidation of crucial metabolic mechanisms involved in heat response in *P. radiata*, as well as the identification of thermotolerance metabolic biomarkers (L-phenylalanine, hexadecanoic acid, and dihydromyricetin), crucial metabolites which can reschedule the metabolic strategy to adapt to high temperature.

## Introduction

As a consequence of climate change, the intensity and frequency of extreme weather events, such as heat waves, are projected to increase, these being one of the major global risks (EEA, 2012). Heat waves (when the temperature rises at least 5°C above normal values) can have a short duration (about a few days). Although plants are known to be able to respond to altered climatic conditions(e.g., Schworer et al., 2014), in woody plants, and specifically the response and heat adaption rate have been barely studied. In forest ecosystems, climate change could have significant implications in timber production (Martinich et al., 2017). Therefore, in order to reach the future demand for wood products, it is necessary to focus research in improving the production, health, and performance of the commercially valued forest species, such as *Pinus radiata*, in future scenarios of increased temperature.

In plants, it has been reported that high temperature has negative effects in various physiological processes such as photosynthesis, primary and secondary metabolism, water relations, or lipid metabolism (Xu et al., 2006). Specifically, it is known that heat stress generates damage to the cell membrane, overproduction of reactive oxygen species (ROS), senescence, inhibition of photosynthesis, and cell death. However, plants have designed specific protection mechanisms to minimize and repair the damage caused by high temperatures in order to cope with heat waves that they will inevitably face during their lifespan.

When a plant perceives environmental stress, multiple signaling cascades are activated. These include the intricate cross-talk between the different plant hormones and other signaling pathways involving kinases and phosphatases, calcium, ROS, and lipids. Previous studies in *P. radiata* showed salicylic acid (SA) and abscisic acid (ABA) as crucial factors in the initial response to heat stress (Escandón et al., 2016), probably due to the rush of the plant to quickly regulate stomatal closure as observed in other species (Acharya and Assmann, 2009). Other phytohormones such as cytokinins (CKs) and indolacetic acid (IAA) seem to be more important for the acclimation and recovery of the plant (Skalák et al., 2016). Nevertheless, how these hormones interact and trigger changes in the plant metabolome to deal with heat stress is still unknown.

Environmental stress results in a reorganization of the metabolism in order to assure homeostasis, which is often accomplished by maintaining essential metabolism and synthesizing metabolites with stress-protective and signaling properties (Fernández de Simón et al., 2017). Changes in secondary metabolism are usually triggered by environmental stresses (Routaboul et al., 2012; Fernández de Simón et al., 2017) such as extreme temperatures, salinity, water availability, or high light intensity, being involved in most plant adaptive responses in combination with signaling mechanisms regulated by plant hormones. Phenolic compounds are one of the most important classes of secondary metabolites in plants as they play important roles in the response to high-temperature stress. Specifically, high-temperature stress promotes the production of phenolic compounds such as flavonoids, phenylpropanoids, anthocyanins, and lignins which are related to the suppression of stress-induced oxidation of most cell molecules (Wahid et al., 2007).

Lipid metabolism is also altered by high-temperature stress as a basic mechanism to control membrane fluidity, cell signaling, and movement of substances (Falcone et al., 2004). According to Grover et al. (2000) and Umesha (2005), the accumulation of highly saturated fatty acids might confer tolerance to high-temperature stress by means of reducing structural membrane fluidity when this is increased by environmental warmth.

Metabolomics profile analyses can be considered an important diagnostic tool for verifying the physiological responses of plant species to environmental changes and to understand the mechanisms behind the complex biological response (Gibbons et al., 2015; Zhang et al., 2015; Meijón et al., 2016), providing a snapshot of the physiological status of the plant in response to an environmental stress (Gibbons et al., 2015; Patel and Ahmed, 2015; Zhang et al., 2015). The feasibility of metabolome analysis for biomarker discovery relies on the assumption that metabolites are important players in biological systems (Monteiro et al., 2013) and stress situations cause drastic changes of metabolomics pathways, which are not new concepts (Shulaev et al., 2008; de Leonardis et al., 2015; Pascual et al., 2017). Currently, using MS-based platforms and combining different analytical technologies, it is possible to increase metabolome coverage. The use of GC-MS technique allows measuring the most of the primary metabolites, while LC-MS provide a better coverage of large hydrophobic metabolites predominant in secondary metabolisms (Doerfler et al., 2013). Both techniques together can assure a better metabolome, allowing the elicitation of a complete view of metabolic dynamics involved in the heat response of *P. radiata*. However, these kinds of studies also require a system biological approach using bioinformatics tools to understand their implications for cell function and to attach the missing connections between molecules and plant physiology (Bruggeman and Westerhoff, 2007; Meijón et al., 2016).

In this study, *P. radiata* plants were exposed to high temperatures aiming to mimic future scenarios of increased warmth. Metabolome and physiological data were analyzed comprehensively using a multivariable approach combining both sets of data. The analysis revealed the dynamic behavior of the metabolic and signaling transduction pathways, as well as connections among the pathways. Classical physiological measurements combined with cutting-edge technologies such as mass spectrometry-based analytical procedures for characterizing the variations in the metabolome revealed key metabolites related to high-temperature response in *P. radiata.* These key metabolites have a possible use as biomarkers in *P. radiata* and other species, due to the similarity of metabolites and basic metabolic pathways between very different species, while proteins, genes, and mRNAs are diversified from one species to another (Peng et al., 2015). Altogether, this work provides a deep knowledge of the response and acclimation process to heat stress in *P. radiata*, as well as the selection of possible universal thermotolerance biomarkers or crucial metabolites which can be further considered by breeders and forest managers.

## Materials and Methods

### Plant Material and Experimental Design

The assay was conducted in a climate chamber under controlled conditions (Fitoclima 1200, Aralab). One-year-old *P. radiata* seedlings (plant size about 33 ± 4 cm) in 1-dm^3^ pots (blond peat:vermiculite, 1:1) were kept under a photoperiod of 16 h (400 μmol m^-2^.s^-1^) at 25°C and 50% relative humidity (RH), and 15°C and 60 % RH during the night period. The plants had been previously acclimated over a 1-month period inside the climate chamber, being watered with nutritive solution (NPK, 5:8:10).

Control plants (C) were collected before starting the heat exposure and were maintained at 25°C for the duration of the trial. Heat exposure treatment began with an increasing temperature gradient from 15 to 40°C over 5 h and maintained for 6 h. This experimental procedure was repeated for 5 days. Sampling was performed at: 3 h after 40°C was reached on day 1 (T1/2) and at the end of the 6-h heat exposure on day 1 (T1), day 2 (T2), day 3 (T3), and day 5 (T5). Plants were watered every day to 80% FC (full capacity) and weekly fertilized with a nutritive solution (NPK, 5:8:10). Plants of each treatment were allowed to recover for 1 month under the control conditions. Recovered plants (R) represent an intermediate exposure time (T3 recovered plants) because there was no significant difference (at the morpho-physiological level) between recovered plants exposed to the different heat exposures. This experimental design aimed to cover the entire stress sensing–response–adaption process, increasing the density of analysis at short-term (T1/2, T1, T2), longer exposures (T5), and recovered plants (R), complementing previous short-term response (C, T1, and T3) analysis (Escandón et al., 2017).

Mature needles from each plant (16 plants/exposure) were sampled, cleaned with a moistened cloth, and immediately frozen in liquid nitrogen until metabolites were extracted. Pools of 3 plants for each biological replicate were performed. Data of physiological measurements – electrolyte leakage (EL), relative water content (RWC), maximum quantum efficiency of PSII (Fv/Fm), quantum yield of photosystem II photochemistry (φPSII), malondialdehyde content (MDA), proline content, starch content, total soluble sugars (TSS), chlorophyll a content (Chla), chlorophyll b content (Chlb), and carotenoid content (Carot) – and fitohormone data – SA, indol-3-acetic acid (IAA), ABA, zeatin riboside (ZR), dihydrozeatin riboside (DHZR), gibberellin 7 (GA_7_), jasmonic acid (JA), gibberellin 9 (GA_9_), isopentenyl adenosine (iPA), isopentenyl adenine (iP), and castasterone (BK) – were taken from Escandón et al. (2016) for multivariable and integrative analysis.

### Metabolite Extraction

Metabolite extraction was performed according to Valledor et al. (2014a) using 100 mg of needle fresh weight. Briefly, samples (C, T1/2, T1, T2, T3, T5, R) were ground in liquid nitrogen and 600 μL of cold (4°C) metabolite extraction solution – Methanol:Chloroform:H_2_O (2.5:1:0.5) – was immediately added to each tube. Then, samples were centrifuged at 20,000 *g* for 4 min at 4°C and the supernatant transferred to new tubes. Finally, 800 μL of Chloroform:water (1:1) were added and the tubes vortexed and again centrifuged at 20,000 *g* for 4 min at 4°C. Two layers formed: an upper aqueous layer, containing the polar metabolites and a lower organic layer, containing the non-polar. Both fractions were transferred to new tubes and dried in a speed vac.

### Polar Metabolite Identification and Quantitation Using LC-Orbitrap-MS Analysis

The polar fraction of each sample was analyzed twice on an LC-Orbitrap-MS, first in positive ion mode and then in negative. A Dionex Ultimate 3000 (Thermo Fisher Scientific, United States) UHPLC was used and a LC-Orbitrap LTQ XL-MS system (controlled by Xcalibur version 2.2, Thermo Fisher Corporation) was run according to the procedure described in Escandón et al. (2017). The resolution and sensitivity of the Orbitrap were controlled by the injection of a mixed standard after the analysis of each batch, and the resolution was also checked with the aid of lock masses (phthalates). Blanks were also analyzed during the sequence.

LC-Orbitrap-MS raw data were processed and compared using MZmine software version 2.10 (Pluskal et al., 2010). MS1 spectra were filtered establishing a noise threshold at 5.5E^03^ and minimum peak height at 6E^03^ with a minimum time peak of 0.15 min. Peaks were smoothed and deconvoluted by using a local minimum search algorithm (98% chromatographic threshold, minimum retention range 5 min, minimum relative height of 90%, and minimum ratio top/edge of 1.2). Chromatograms were aligned using the RANSAC algorithm with a tolerance of 5 ppm of and 1.0 min retention time. Normalized peak areas were used for quantification, and their values were log transformed before statistical analyses (Supplementary Table S1a).

The individual peaks were identified following different approaches; the first step was performed against an *in-house* library (>100 compounds) and manual annotation considering m/z and retention times. In the second step, masses were assigned using the KEGG, PubChem, METLIN, MassBank, HMDB, and Plantcyc databases as reported by Escandón et al. (2017) with built-in MZmine plugins with a 5 ppm threshold and considering as “identified” beyond doubt those metabolites that were defined after the comparison to our standard compound library or by a matching of MS/ MS to the small number of plant compounds for which their MS/MS is available in public databases (Meijón et al., 2016); and as “tentatively assigned” those with molecular ions with exact masses corresponding to identified metabolites in databases. Metabolite identification against our library was confirmed by retention time (RT), mass, isotopic pattern, and ring double bound parameters. Supplementary Data S1 includes the detailed interpretation of experimental MS/MS spectra which support our tentative identifications of the candidate metabolites that were not identified beyond doubt in the first term.

### Non-polar Metabolite Identification and Quantitation Analysis

Non-polar metabolites were derivatized with 295 μL tert-methyl-Butyl-Ether (MTBE) and 5 μL of trimethylsulfonium hydroxide (TMSH) for 30 min at room temperature. The tubes were centrifuged for 3 min at 20,000 *g* to remove insoluble particles before transferring the supernatants to GC-microvials. GC-MS measurements were carried out following a previously developed procedure (Valledor et al., 2014b) on a triple quad instrument (TSQ Quantum GC; Thermo, United States). The mass spectrometer was operated in electron-impact (EI) mode at 70 eV in a scan range of m/z 40–600. Metabolites were identified based on their mass spectral characteristics and GC retention times through comparison with the retention times of reference compounds in an in-house reference library and the current version of Golm Metabolome Database (Hummel et al., 2007) using LC-Quant software (Supplementary Table S1b).

### Quantitative Real-Time PCR of Selected Genes

RNA was extracted from 100 mg of needle fresh weight as described by Valledor et al. (2014a). cDNA was obtained from 1,000 ng of RNA using the RevertAid kit (Thermo Scientific, United States) and random hexamers as primers following the manufacturer’s instructions. Later, qPCR reactions were performed in a CFX Connect Real Time PCR machine (Bio-Rad) with SsoAdvanced Universal SYBR Green Supermix (Bio-Rad, United States); three biological and two analytical replicates were performed for each treatment.

*ACTIN* (*ACT*), *RIBOSOMAL PROTEIN 18S, GLYCERALDEHYDE 3-212 PHOSPHATE DEHYDROGENASE* (*GAPDH*), and *UBIQUITINE* (*UBI*) genes were tested as endogenous control employing geNorm following the criteria of Hellemans et al. (2007). ACT and UBI were the most stable and consequently selected as endogenous genes. Normalized Relative Quantities (NRQ) and Standard Errors of RQ were determined according to Hellemans et al. (2007). Primers were designed using transcript sequences available in a *P. radiata* in-house database obtained from RNA-Seq data (unpublished results). Detailed information about the primers used for qPCR experiments is available in Supplementary Table S2.

### Statistical and Bioinformatics Analysis

Five biological replicates were used for metabolites and physiological parameter statistical analysis. The procedures were conducted with the R programming language running under the open-source computer software R v2.15.2 (R Development Core Team, 2015) and RStudio (RStudio Team, 2015). Metabolome datasets were pre-processed following the recommendations of Valledor et al. (2014c). In brief, missed values were imputed using a k-nearest neighbors approach, and variables were filtered out if they were not present in all replicates of one treatment or in at least 45% of the analyzed samples. Data was transformed following a sample-centric approach followed by log transformation. Centered and scaled values (z-scores) were subjected to multivariate analysis and Heatmap clustering. The calculation of the number of common metabolites for all combinations of treatments and unique metabolites in a single treatment was performed using core functions of R.

Metabolomics pathways of each metabolite (Supplementary Table S3a) were searched against KEGG pathway maps (KEGG Mapper, Kanehisa et al., 2012) and *p*-values of each metabolomics pathways (Supplementary Table S3b) in MBROLE 2.0 (López-Ibánez et al., 2016). Heat mapping was carried out using the Manhattan distance method to group metabolites in different KEGG pathways with an MBROLE FDR correction of less than 0.05. Multivariate analysis of metabolites and physiological parameters (Supplementary Table S4) were conducted with mixOmics (Lê Cao et al., 2009) using Principal Component Analysis (PCA), Sparse Partial Least Squares (sPLS), and network analyses. The normalization of the datasets was performed before combining them. sPLS algorithm was used to find correlations between predictor (metabolites matrix) and response variables (physiological parameters) and its graphic representation in the network analysis. Network topology was defined after applying sPLS regression using the function network() of the mixOmics package and filtered (only edges equal or higher than |0.60| were maintained) in Cytoscape v.3.3.0 (Cline et al., 2007). Univariate analyses were conducted: one-way ANOVA, *p* < 0.05 for metabolites and physiological parameters and Student’s *t*-test, *p* < 0.1 for qPCR analysis. Graphics were plotted employing ggplot2 (Wickham, 2009) and pheatmap (Kolde, 2015).

## Results

### Characterization of the Metabolome During Heat Treatment

The combination of LC-Orbitrap-MS for polar and GC-MS for non-polar metabolites allowed the reliable quantification of a total of 2,287 ions based on the obtained m/z and retention times (Supplementary Table S1). These different ions can be considered different metabolites since each combination of m/z and retention time should be unique for each metabolite and spatial conformation. The fusion of a customized searching/identification algorithm based on in-house and public databases resulted in the unequivocal identification of 41 ions (identical matches to our compound library) and 747 ions that were tentatively assigned after comparing its very accurate mass against reference compound databases (Supplementary Table S1). The combination of both ionization modes (GC-MS and LC-Orbitrap-MS) gave a broad characterization of the pine metabolomes during heat-induced response, which covered most of the primary and secondary metabolism pathways.

**FIGURE 1:**
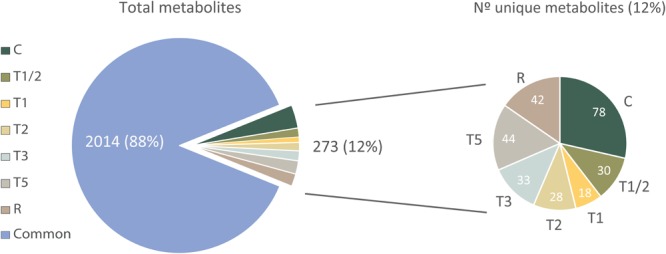
Total metabolite diagram: Qualitative common metabolites (2,014) and unique metabolites (273) for each treatment. C, control; R, recovered; T1/2, 3 h after 40°C on day 1; T1, 6-h heat exposure on day 1; T2, day 2; T3, day 3; T5, day 5; common, qualitative common metabolites in at least two treatments.

Metabolome analysis showed that most of the metabolites are present in all treatments or at least in two different treatments (880 or 2,014 metabolites, respectively, “common” in **Figure 1** and Supplementary Table S5). Conversely, a total of 273 metabolites were identified only in one of the treatments (**Figure 1**), Control (C) being the sample that showed the highest number of characteristic metabolites (78), followed by T5 (44) and R (42). Otherwise, T5 and C were the treatments that shared a greater number of metabolites (106 metabolites; Supplementary Table S5).

**FIGURE 2:**
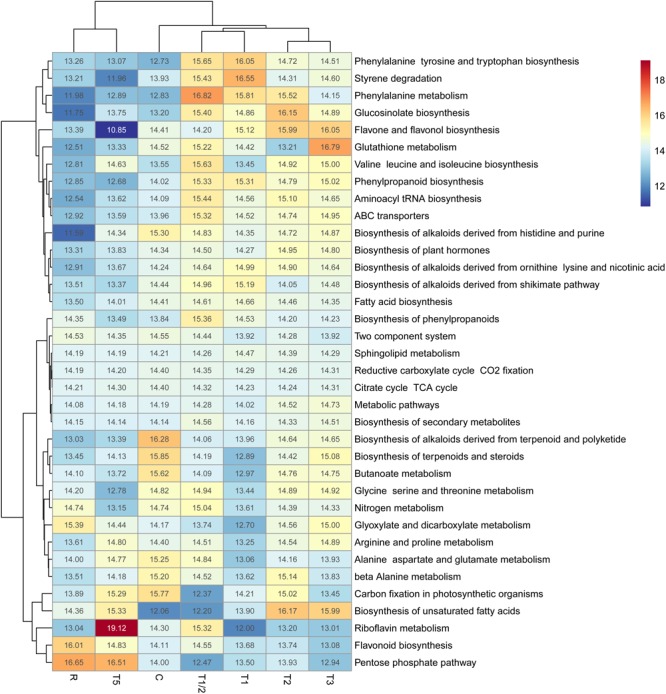
Heatmap-clustering analyses of KEGG pathways considering only the pathways that exceed MBROLE FDR-correction (*p* < 0.05). Numbers inside the boxes indicate normalized abundance of each pathway (as a percentage) calculated as the sum of all identified/assigned metabolites within each pathway according to KEGG pathway. C, control; R, recovered; T1/2, 3 h after 40°C on day 1; T1, 6-h heat exposure on day 1; T2, day 2; T3, day 3; T5, day 5.

The complexity of the metabolome data was reduced by focusing on the specific pathways in relation to the relative abundance of the metabolites identified. Heatmap-clustering analysis (**Figure 2** and Supplementary Figure S1), distinguished four different groups in relation to metabolic pathways identified: C, T1/2-T1, T2-T3, and T5-R group that is highly separated of C and shorter-term treatments.

On the other hand, the clustering based on the profile of KEGG pathways did not show any clear trend, each group presenting a different pathway profile across the sample times, e.g., glucosinate biosynthesis and flavone and flavonol biosynthesis increased their activities in treatments T1/2, T1, T2, or T3; conversely, pentose phosphate pathway and flavonoid biosynthesis showed the maximum in T5 and/or R. Other pathways, such as the biosynthesis of unsaturated fatty acids increased the activity in T2, T3, and T5 or only in the longer-term exposure (T3 and T5) as arginine and proline metabolism pathways. In contrast, pathways related with phenylalanine metabolism enhance their activity during the stress but decrease at T5 (at the levels of C and R). Lastly, riboflavin metabolism showed a substantial increase only in T5 and glutathione metabolism showed a fluctuating tendency, highlighting its maximum in T3.

These results seem to underlie two different types of heat-response in *P. radiata* (shorter-term and longer-term response) where different pathways are necessary at a different time of stress.

### Shorter-Term and Longer-Term Responses to Heat Stress Were Confirmed by Integrative Analysis of Metabolome and Physiological Datasets

**FIGURE 3:**
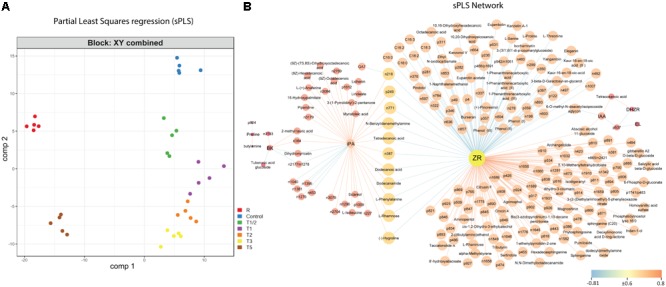
Multivariate analysis of metabolome, and physiological parameters in needles during heat treatment. **(A)** Classification of the different samples according to sPLS. Components 1 and 2 allowed the clustering of treatments analyzed according to shorter-term (T1/2, T1, T2, and T3) and longer-term (T5 and R) heat-stress responses. **(B)** Interaction networks constructed after sPLS analysis using metabolites as the predictor matrix and physiological measurements as the response matrix. Edge color represents the correlation value. Only those correlations equal or higher, in absolute value, than 0.6 are shown. Color nodes reflect the amount of control that this node exerts over the interactions of other nodes in the network (higher control = lighter color). EL, electrolyte leakage; ZR, zeatin riboside; iPA, isopentenyl adenosine; IAA, indol-3-acetic acid; DHZR, dihydrozeatin riboside; BK, castasterone; GA7, gibberellin 7; C, control; R, recovered; T1/2, 3 h after 40°C on day 1; T1 6-h heat exposure on day 1; T2, day 2; T3, day 3; T5, day 5.

To simplify the dimensionality of the results and integrate metabolome data with physiological datasets, sPLS (**Figure 3A** and Supplementary Table S6) and PCA (Supplementary Figure S2 and Supplementary Table S7) analyses were used. sPLS and PCA scores revealed a trajectory of the different sampling times by the combination of the two main components. Recovered (R) and long-term exposed (T5) plants were separated from the other treatments by the first component, which seems to be gathering the variance related to long-term stress adaption, while the second component, considering top correlated variables (Supplementary Figure S2 and Supplementary Table S6) is related to heat-response/tolerance. This component revealed differences between Control-Recovered and heat-treatments.

Altogether, multivariate results provided hints about two different responses to stress. First, an initial response to stress (shorter-term response), involves increased ABA and SA activities in samples T1/2, T1, T2, and T3 (**Figure 3A**, Supplementary Table S6a, Comp 1). Secondly, adaptive mechanisms, such as TSS and ZR, seem to be involved in the distinction between T5 and T3, as well as between the recovered and control plants according to sPLS analysis (**Figure 3A**, Supplementary Table S6a, Comp 1).

The interaction networks built from this analysis (**Figure 3B** and Supplementary Table S6) showed a complex correlation between different metabolites and hormones and physiological parameters. The important role of CKs was demonstrated during heat response, as well as the links of these hormones with different compounds of the metabolome and physiological parameters in response to high temperatures. Two main nodes in the constructed network were detected: ZR and iPA nodes. The ZR node was negatively correlated with numerous saturated and unsaturated fatty acids (C16:0 and C18:0 with their unsaturated forms), flavonoids (including kandelin A-1 or eujambolin), amino acids (such as L-proline and L-serine), and L-phenylalanine (key metabolite in several pathways, such as phenylalanine metabolism, phenylpropanoid biosynthesis and biosynthesis of plant hormones). The presence of L-proline in the network indicates the relevance of the accumulation of this amino acid in longer-term response (T3 and T5) to high temperatures in *P. radiata*, as it has already been showed in Escandón et al. (2016) and confirmed by the increased activity of its metabolism (**Figure 2**, arginine and proline metabolism). Additionally, ZR was positively correlated with fatty acids involved in sphingolipid metabolism, terpenoids (including abscisic-alcohol 11-glucoside) and other secondary metabolites: dihydrokaempferol (related to flavonoid biosynthesis) and *cis*-1,2-dihydro-3-ethylcatechol (implicated in the degradation of aromatic compounds). In this node, it is also important to note that the physiological parameter, EL, appeared negatively correlated by an unknown metabolite (p537) to IAA and DHZR which, in turn, were also positively linked to tetracosanoic acid (saturated fatty acid, C24:0). This confirms the relevance of these hormones (Du et al., 2013; Černý et al., 2014) and fatty acid signaling (Los and Murata, 2004) to repair membrane damage related to heat stress. On the other hand, iPA was positively correlated with fatty acids, such as tetradecanoic acid and (9Z)-octadecenoic acid, as well as metabolites involved in diterpenoid biosynthesis (sclareol); and this hormone was negatively correlated with compounds involved in flavonoid biosynthesis, such as dihydromyricetin.

Interestingly, the analysis of the network dynamics and particularly the quantitation of the represented variables showed a two-step response, with T3 as the transition point between shorter-term and longer-term responses (Supplementary Movie S1). This observation is consistent with the conducted sPLS and PCA analyses. This behavior can be considered an adaptive mechanism to cope with rapid environmental changes that occur daily (i.e., sun heat following a rainy period). In this case, plants require a mechanism to quickly overcome the first impact of stress; however, the physiology should return to ideal values after the removal of the stress factor in order to achieve an optimal energetic balance. A previous work (Escandón et al., 2016) showed that photosynthetic state was only slightly affected in the first impact to heat stress (T1/2, T1 and T2), recovered to control levels in T3, and even improved on T5, probably related to the beginning of the acclimation process; while lipids peroxidation analysis showed a slight accumulation of MDA in shorter-term exposures (T1/2, T1 and T2) prior to the activation of acclimation mechanisms. On the other hand, if the stress persists the plant must adapt to the new situation with the cost of reducing its growth and reproductive capacity compared to an ideal situation (Bradford and Hsiao, 1982).

### Using Metabolomics to Explore Possible Thermotolerance Biomarkers

The metabolome analysis revealed the essential role of specific metabolites (**Figure 3**) and pathways (**Figure 2** and Supplementary Figure S1) in relation to high-temperature adaptation in *P. radiata*.

**FIGURE 4:**
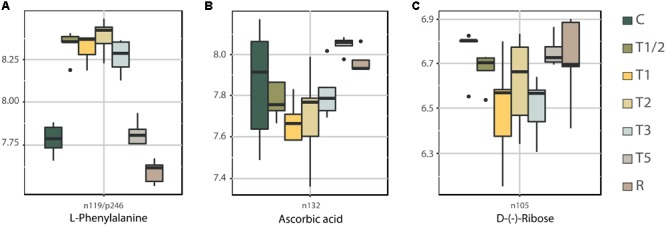
Time-course accumulation of key metabolites involved in phenylalanine metabolism (L-phenylalanine) **(A)**, glutathione metabolism (Ascorbic acid) **(B)**, and pentose phosphate pathway and ABC transporters (D-Ribose) **(C)**. Box plot representation of the LOG transform data (Supplementary Table S4). The ID of each metabolite is upper the identification by in-house database. C, control; R, recovered; T1/2, 3 h after 40°C on day 1; T1, 6-h heat exposure on day 1; T2, day 2; T3, day 3; T5, day 5.

The accumulation of three different metabolites belonging to pathways with higher significant changes across high-temperature treatments is showed in **Figure 4**. One of these three key compounds is L-phenylalanine which participates in numerous pathways (Meyermans et al., 2000; Wittstock and Halkier, 2000; Teufel et al., 2010; Tzin and Galili, 2010; Yoo et al., 2013), such as phenylalanine metabolism, phenylpropanoid biosynthesis, phenylalanine, tyrosine and tryptophan biosynthesis, glucosinolate biosynthesis, and biosynthesis of plant hormones, most of them highly related/interconnected. Its accumulation profile reflected a level increase from T1/2 to T3 (**Figure 4A**), decreasing to control levels in T5 and reaching the lowest accumulation values in R. Phenylpropanoids and flavonoids play a key role in protecting plants against abiotic stress, largely by inhibiting the formation of ROS through a number of different mechanisms (Mierziak et al., 2014). The increment of L-phenylalanine in the shorter treatments may be related to the need to cope with the production of ROS. The decrease in L-phenylalanine in T5 and R could be related to the increased activity of *PHENYLALANINE AMMONIA LYASE* (*PAL*; crucial enzyme in phenylpropanoid pathway), which transforms L-phenylalanine into *trans*-cinnamic acid (Rivero et al., 2001). The activity increase of this enzyme is considered one of the most important ways of cell acclimation against stress in plants (Levine et al., 1994; Leyva et al., 1995; Rivero et al., 2001).

Other pathway highlighted by heatmap-clustering (**Figure 2** and Supplementary Figure S1) was the glutathione metabolism, which showed the highest accumulation in T3. Ascorbic acid is key in this pathway; however, it showed decreased levels in short-term treatments (**Figure 4B**), reaching the highest values in T5 and R. Ascorbic acid and glutathione are both antioxidants, which are crucial for plant defense against oxidative stress (Noctor and Foyer, 1998). The decrease of ascorbic acid in shorter treatments could be explained by their use in this defense against oxidative damage.

D-(-)-ribose (**Figure 4C**), which participates in pentose phosphate and ABC transporters pathways, showed a decline in shorter-term, recovering the control values in T5 and R. This may indicate that plants reduce their metabolism until T3, recovering the activity in T5 and R when the plants are adapted. Although sugars play an important role against heat stress in many species (Wahid et al., 2007), in *P. radiata* it has been showed that the total amount of soluble sugars tends to decrease in the first moments of stress (Escandón et al., 2016). This may be because plants maintains growth patterns although they are driven by consuming carbohydrates reserves (Mitchell et al., 2013; Escandón et al., 2016).

**FIGURE 5:**
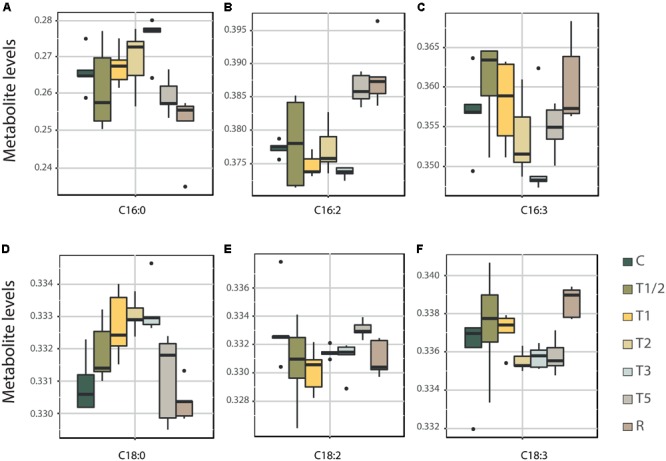
Levels of key metabolites of unsaturated fatty acids metabolism across high-temperature treatments. C16: Saturated fatty acid **(A)** and their unsaturated fatty acids C16:2 **(B)** and C16:3 **(C)**. C18: Saturated fatty acid **(D)** and their unsaturated fatty acids C18:2 **(E)** and C18:3 **(F)**. Box plot representation of LOG transform data for the total content of each fatty acid C16 or C18, respectively. C, control; R, recovered; T1/2, 3 h after 40°C on day 1; T1, 6-h heat exposure on day 1; T2, day 2; T3, day 3; T5, day 5.

Under high temperatures, plants alter lipid composition, causing membranes to become more fluid and thus interrupting membrane processes (Falcone et al., 2004). High-temperature-tolerant plants show an increased presence of saturated fatty acids to counteract increased fluidity during heat stress (Grover et al., 2000; Umesha, 2005). Levels of candidate metabolites related to biosynthesis of unsaturated fatty acids pathway are represented in **Figure 5** including both saturated fatty acids (**Figures 5A,D**) and unsaturated fatty acids (**Figures 5B,C,E,F**). Saturated fatty acids, hexadecanoic acid (C16:0, **Figure 5A**) and C18:0 (**Figure 5D**), shared an increasing tendency in short-term response, starting to fall in T3 (in the case of C16:0) or earlier in T2 (C18:0). In both, the lowest accumulation values were reached in R. This saturated fatty acids raise is consistent with the hypothesis by Grover et al. (2000), which indicates the plants increase saturation until seems to be already acclimated to the heat stress.

In the case of unsaturated fatty acids, they showed different patterns depending on the studied fatty acid. C16:2 and C18:2 showed a slight decrease in short-term response (**Figures 5B,E**), conversely, C16:3 and C18:3 (**Figures 5C,F**) presented increased levels in T1/2 and T1. In longer-term response, the most common tendency is the recovery at the control values (**Figures 5B,C,E**) even surpassing them, except for C18:3 where this only occurs in R (**Figure 5F**). This differential accumulation patterns seems to evidence the different roles of each unsaturated fatty acid during heat-stress response.

**FIGURE 6:**
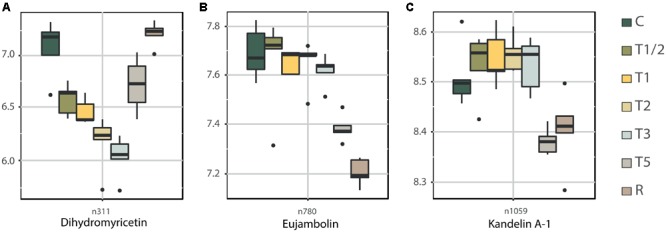
Levels of key metabolites related to flavonoid biosynthesis across high-temperature treatments: Dihydromyricetin **(A)**, eujambolin **(B)**, and kandelin A-1 **(C)**. Box plot representation of the LOG transform data (Supplementary Table S4). ID of the three metabolite, previously tentatively identified using public database, was validated by the interpretation of their MS/MS spectra (Supplementary Data S1). C, control; R, recovered; T1/2, 3 h after 40°C on day 1; T1, 6-h heat exposure on day 1; T2, day 2; T3, day 3; T5, day 5.

Flavonoids related pathways are also crucial elements according to heatmap-clustering analysis (**Figure 2** and Supplementary Figure S1). Bibliography indicate that flavonoids and anthocyanins are essential compounds to prevent and protect the plants against different abiotic and biotic stress (Dao et al., 2011; Zhang et al., 2012). Results showed that dihydromyricetin (**Figure 6A**), key metabolite in flavonoid biosynthesis pathway, continuously decreases its levels until T3, to then begin its accumulation, reaching the control values in R. On the contrary, flavonoids eujambolin and kandelin A-1 (**Figures 6B,C**), showed the lowest accumulation in T5 and R. Eujambolin presented a decreasing tendency in shorter treatments, showing a strong decrease in T5 and later in R. Conversely, kandelin A-1 showed an increase in shorter treatments in relation to control, displaying a strong decrease in T5 and R.

Dihydromyricetin (along with dihydroquercetin and dihydrokaempferol) is a dihydroflavonols implicated in the synthesis of anthocyanidins (Falcone Ferreyra et al., 2012). While, kandelin A-1 is a proanthocyanidin which are synthesized as oligomeric or polymeric end products of one of several branches of the flavonoid pathway, which shares the same upstream pathway with anthocyanins (He et al., 2008). Anthocyanins are usually accumulated during heat stress in vegetative tissues (Wahid and Ghazanfar, 2006) in order to decrease the transpirational losses caused by lower osmotic potential of the leaf (Chalker-Scott, 2002). However, the complexity of its metabolism and the high number of these compounds makes their study difficult.

**FIGURE 7:**
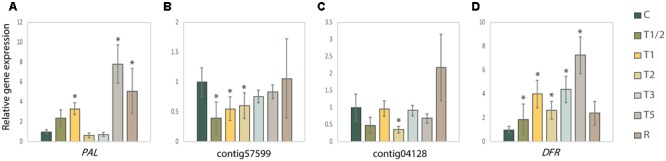
Analysis of the relative quantity (RQ) measured by RT-qPCR of selected genes in highlight pathways related to high-temperature response of *Pinus radiata*. **(A)** *PHENYLALANINE AMMONIA LYASE* (*PAL*) involved in phenylpropanoid biosynthesis; **(B)** *DESATURASE* (*contig57599*) and **(C)** *DESATURASE* (*contig04128*) in unsaturated fatty acids metabolism; **(D)** *DIHYDROFLAVONOL 4-REDUCTASE* (*DFR*) in anthocyanins and proanthocyanins biosynthesis. Expression levels are shown regarding the Control and were normalized using *ACT* and *UBI* as housekeeping genes. Error bars show the SE of normalized RQ for each gene and each sample scaled to the control. Asterisks (^∗^) denote statistically different values compared to the corresponding control group (according to Student’s *t*-test, *p* < 0.1). C, control; R, recovered; T1/2, 3 h after 40°C on day 1; T1, 6-h heat exposure on day 1; T2, day 2; T3, day 3; T5, day 5.

Gene expression of *PAL* (**Figure 7A**), two *DESATURASE* (*DES*, **Figures 7B,C**) and a central gene involved in anthocyanins and proanthocyanins biosynthesis pathways, *DIHYDROFLAVONOL 4-REDUCTASE* (*DFR*, **Figure 7D**) (Ayabe et al., 2010; Katsu et al., 2017) were analyzed in order to confirm the possible role of these elements as biomarkers and validate the relevance of L-phenylalanine, fatty acid and flavonoid metabolism in the high-temperature response of *P. radiata*.

These data showed that *PAL* (**Figure 7A**) increases its expression in the first moments to stress (T1/2 and T1), returning to the control values in T2 and T3. In T5 and R its expression increases drastically, confirming the pattern of decrease of L-phenylalanine (**Figure 4A**) in these treatments by its consumption. In the case of *DES* (**Figures 7B,C**), both genes showed a reduction of the expression in shorter-term treatments: *contig5799* presented the decrease in T1/2, T1 and T2, while *contig04128* only in T2. This reduction is consistent with the increase of saturated fatty acids identified (C16:0 and C18:0, **Figures 5A,D**). Finally, *DFR* (**Figure 7D**) showed an increase of expression across of stress time, reaching a peak in T5 and return to the control values in R. These results are according dihydromyricetin levels quantified (**Figure 6A**), which seems to be being consumed for the production of anthocyanins in response to high-temperature stress.

## Discussion

### Metabolome Characterization: Dynamic of Metabolism Throughout High-Temperature Stress

The plant metabolome responds to an unfavorable environment in a dynamic way, being favored a characteristic type of metabolic pathways at every moment of stress. Depending on the timing, different compounds can be identified, as stress signal transduction molecules, stress metabolism by-products, or molecules that are part of the plant acclimation response (Shulaev et al., 2008). According to this, the results of this work showed that the response of *P. radiata* at high temperatures activates the synthesis of specific metabolites at each time of the stress (**Figure 1**) showing every sample-time a significant number of unique metabolites. Additionally, these results seem to reveal the beginning of the acclimation process in T5 that showed the greatest number of unique metabolites but also the greatest number of shared metabolites with the control plants (106 metabolites, Supplementary Table S4).

In-depth analysis of metabolite accumulation in relation to KEGG pathways (**Figure 2** and Supplementary Figure S1) allowed to confirm the high dynamism of the metabolome in relation to high-temperature response (Escandón et al., 2017). Although two main trends in the KEGG pathways have been identified by heatmap-clustering (**Figure 2** and Supplementary Figure S1), each pathway seem to have a specific role in a particular moment of the stress, seemingly following an orchestrated succession, particularly, in the case of flavonoids related pathways. Thus, phenylpropanoid biosynthesis seems to be activated in the first contact with stress (T1/2-T1), while in T2-T3 the most active route is flavone and flavonol biosynthesis, to finally increase the activity of the general pathway of flavonoid biosynthesis in T5 and R. The importance of the flavonoids across the heat stress is evident. According to bibliography the compounds in this group act as antioxidants, contributing to the adaptation to environmental changes such as cold, high temperatures or irradiation (Gould et al., 2002; Doerfler et al., 2013, 2014; Meijón et al., 2016).

Fatty acids also play a fundamental role in the response to stress in *P. radiata*. Biosynthesis of unsaturated fatty acids is a very active pathway in the longer exposure treatments (T2, T3 and T5), while saturated fatty acid biosynthesis is more relevant in the first moments of exposure to heat (T1/2 and T1). A reduction of unsaturated fatty acid content and increase in saturated fatty acids content has been positively associated with heat tolerance as they counteract the increase in membrane fluidity caused by high temperature (Grover et al., 2000; Larkindale and Huang, 2004). However, this hypothesis has not been fully confirmed since that unsaturated fatty acid are also known to be key elements in heat stress signaling (Königshofer et al., 2008) and to provide other essential characteristics to lipid membrane (Falcone et al., 2004), showing an increase in its accumulation at high temperatures.

In the case of *P. radiata*, biosynthesis of fatty acid is a key mechanism to overcome the fluidization of the membrane until T2 at the beginning of the stress, when the plants had overcome this fluidization. The global increment in saturated fatty acid would be consequence of the increase of the amount of a specific saturated fatty acid (e.g., hexadecanoic acid) or the accumulation of fatty acids with lower numbers of double bonds (the reduction of C18:3 to C18:2 or C18:1; Wang et al., 2017). From T2, plants activate the biosynthesis of unsaturated fatty acids which may be necessary to stabilize photosynthesis (Gombos et al., 1994) or activate signaling (e.g, lipid or calcium signaling) (Königshofer et al., 2008). These observations support the idea of Falcone et al. (2004) that the unsaturation level of lipid membranes also plays an important role in the plant’s ability to tolerate high temperatures, although other characteristics of plants membrane lipids are also likely to be important.

The study of the metabolome across heat stress and recovery, has given the possibility to deepen in the different dynamics of the metabolomic pathways in *P. radiata*, establishing the existence of key pathways in different moments of the stress response.

### Integrative Analysis of Physiological Response and Metabolome Dynamic

This work revealed that high temperature has a complex impact on cell function, suggesting that many and complex processes are involved in heat-resistance processes. However, the use of system-wide approach and integrative bioinformatics tools have allowed the understanding of the molecular basis, the identification of injury mediators, and the characterization of associated biomarkers. Thus, CKs, fatty acid metabolism and flavonoid and terpenoid biosynthesis were revealed as the most relevant pathways related to shorter-term and longer-term response clusters confirmed by multivariate analysis (**Figure 3A**), being ZR and iPA the key hormones that coordinate the different response processes (**Figure 3B**).

Some of the secondary metabolites with a higher loading in the shorter-term and longer-term response cluster included dihydrokaempferol, *cis*-1,2-dihydro-3-ethylcatechol and dihydromyricetin, which are all crucial elements in flavonoid metabolism. Flavonoids are a biologically and chemically diverse group widely represented in plants. Their diversity and multi-functionality demonstrate their importance in plants. Terpenoids are the other significant group of secondary metabolites identified in relation to the clusters, which are produced by a variety of plants and particularly in conifers (Zulak and Bohlmann, 2010). These volatile compounds are emitted by plants and play an important role in the interaction with their environment (Tholl, 2015). The best known and most studied group of terpenoids is the sesquiterpenoid plant hormone ABA, the central element in the plant stress response (McCourt et al., 2005; Raghavendra et al., 2010). Abscisic alcohol 11-glucoside, the glycosylated form of ABA, was also positively correlated to ZR as a key element in the network (**Figure 3B**). Both groups of secondary metabolites, flavonoids and terpenoids, are important in plant growth, development, and response against biotic and abiotic stress (Alder et al., 2012), as well as in plant adaptation to variable environmental conditions (Kliebenstein, 2004). Plasma membrane fluidity has been described to be an important temperature sensor in plants that appears to lie upstream of the unfolded protein response (Wu et al., 2012). Increased membrane fluidity appears to open calcium channels in the plasma membrane and the resultant inflow of calcium triggers signaling cascades, including an H_2_O_2_ burst (Königshofer et al., 2008), which activates the heat-stress response. In fact, fatty acids are the most prominent group of compounds revealed in the heat-stress response network, after CKs.

Integrated physiological and metabolome analysis across high-temperature treatments (Supplementary Movie S1) in *P. radiata* emphasizes the complex dynamics of the metabolome in response to heat stress and suggests the existence of a turning point (T3) at which *P. radiata* plants changed from an initial stress response (shorter-term response) to an acclimation one (longer-term response). The video highlights how the metabolites that are positively related to ZR increase drastically its accumulation in T5 and R. This is regulated by a complex interaction network that involves multiple pathways and groups of compounds where possible biomarkers of thermotolerance processes could be found.

### Evaluation of Proposed Thermotolerance Biomarkers in *P. radiata*

In plants, the concept of biomarker could be defined as “a characteristic that is objectively measured or evaluated as a predictor of plant performance” (Fernandez et al., 2016). The use of biomarkers originated from the field of medicine, but in plants in recent years, many authors have used metabolites as indicators for estimating plant performance under stress conditions (Quistian et al., 2011; Degenkolbe et al., 2013; Nam et al., 2015; Obata et al., 2015). One of main goals in this study is to find metabolic markers of high-temperature tolerance, potentially useful for *P. radiata* breeding programs.

L-phenylalanine, hexadecanoic acid (C16:0), and dihydromyricetin were confirmed as the three strongest biomarkers between the proposed candidates related to the results of this work. Their biological relevance, statistical strength in integrative analysis, and accumulation profile across the samples times analyzed validate their future use as thermotolerance biomarkers in *P. radiata*.

L-phenylalanine seems to be one of the clearest candidates. L-phenylalanine is crucial in numerous KEGG pathways which changed significantly during stress, particularly in shorter-term treatments (e.g., phenylalanine metabolism, phenylpropanoid biosynthesis, phenylalanine, tyrosine and tryptophan biosynthesis, glucosinolate biosynthesis, and biosynthesis of plant hormones). In addition, integrative analysis of metabolome and physiological measurements showed its relevance as a key compound in the network related to high-temperature response (**Figure 3B**). L-phenylalanine showed a direct correlation with the CKs associated with the trigger of response (negatively with ZR and positively with iPA). Old studies (Dedio and Clark, 1971; Deikman and Hammer, 1995) have already seen how the application of CKs stimulated flavonoids pathways in which L-phenylalanine is involved (flavonoids are synthesized by the phenylpropanoid metabolic pathway), such as the production of isoflavone and anthocyanins synthesis. More recently, Angelova et al. (2001) and Ali and Abbas (2003) have debated the impact of CKs on the accumulation of flavonol glycosides and Hamayun et al. (2015) found out how kinetin modulates isoflavone contents under salinity stress. Furthermore, *PAL* expression analysis (**Figure 7A**) confirms the beginning of the acclimation process in T5 when L-phenylalanine levels (**Figure 4A**) showed a significant decrease, validating the high value of this metabolite as a biomarker.

The second candidate biomarker was chosen within saturated fatty acids, given their importance in stabilizing membrane fluidity during heat stress (Grover et al., 2000; Larkindale and Huang, 2004; Wang et al., 2017). Hexadecanoic acid (also known as palmitic acid or C16:0) increases its accumulation in shorter heat exposures, suffering a decrease in T5 and R, when the plant can be already acclimatized to stress (**Figure 5E**). Moreover, the low expression levels of both *DES* (**Figures 7B,C**) in shorter treatments underline the importance of saturated fatty acids, such as hexadecanoic acid, in the early response to high temperatures. This saturated fatty acid has already been studied by other authors such as Alfonso et al. (2001), Falcone et al. (2004), and Larkindale and Huang (2004) in relation to high-temperatures response. It has been even observed that the thermotolerance of an Arabidopsis mutant deficient in palmitic acid unsaturation is enhanced (Kunst et al., 1989). Furthermore, hexadecanoic is showed as an important element by the performed integrative analysis, appearing interconnected with the master regulator ZR in the network.

The last strong biomarker proposed is dihydromyricetin, also known as ampelopsin. Dihydromyricetin is a flavanonol included in anthocyanin biosynthesis inside flavonoid biosynthesis pathway. Flavonoids and anthocyanins have a photoprotective and antioxidant role (Dao et al., 2011; Zhang et al., 2012). Dihydromyricetin is consumed during anthocyanins synthesis, reducing its accumulation in shorter treatments and reaching the lowest levels in T3 (**Figure 6A**). This is validated by the increased expression of *DFR* across the exposure to high temperatures (**Figure 7D**), showing its maximum expression in T5 when dihydromyricetin begins to be accumulated again. Anthocyanins are produced under a variety of stresses such as UV-B (Valledor et al., 2012), low temperatures (Krol et al., 1995), or salinity (Wahid and Ghazanfar, 2006), although only a few studies have dealt with the effect of high temperatures on anthocyanin accumulation (Wahid and Close, 2007; Correia et al., 2014; de Leonardis et al., 2015). Dihydromyricetin was also prominent in multivariate and integrative analysis showing a negative correlation with iPA and BK. Cytokinin increases anthocyanin content and the transcript levels of *PRODUCTION OF ANTHOCYANIN PIGMENT 1* (Das et al., 2012) which is according to the dropping levels of dihydromyricetin (anthocyanin precursor) observed in the first impact of the heat stress. Among many other biological functions, anthocyanins are considered the first line of defense against oxidative stress (Gould et al., 2002), scavenging oxygen radicals, and inhibiting lipid peroxidation (Chalker-Scott, 2002; Ling et al., 2007).

## Conclusion

This work shows that high temperatures induced a quick and dynamic change in the metabolome of *P. radiata*, in order to maintain homeostasis and facilitate survival. Integrative study of metabolome across high-temperature exposure and recovery plants allowed reaching a global view of molecular mechanism behind high-temperature response in *P. radiata*, revealing complex interaction networks that involve CKs, fatty acid metabolism, and flavonoid and terpenoid biosynthesis being ZR and iPA the master regulators that trigger the global response. Additionally, novel potential thermotolerance biomarkers such as L-phenylalanine, hexadecanoic acid and dihydromyricetin have been proposed. However, these potential biomarkers need to be validated in further studies in *P. radiata* and their possible universality analyzed in other species.

## Author Contributions

ME, MM, MC, and LV designed and performed the experimental work. ME processed the samples, integrated the datasets, and completed the metabolome analyses. LV and MM performed the mass spectrometry. GP realized the physiological analysis. ME and JP were involved in the preparation of samples. LV and JP helped with statistical analyses. ME and MM wrote the manuscript while MC, LV, JP, and GP supervised the manuscript. All authors read and approved the final manuscript.

## Conflict of Interest Statement

The authors declare that the research was conducted in the absence of any commercial or financial relationships that could be construed as a potential conflict of interest.
