# Supplementary material for: Metabolome Integrated Analysis of High-Temperature Response in Pinus radiata
Source: Front Plant Sci. 2018 Apr 17;9:485. doi: 10.3389/fpls.2018.00485 (PMC5914196; doi:10.3389/fpls.2018.00485)
Supplement: Supplementary file 9 [file Presentation_1.PDF]

Supplementary Material:

Metabolome integrated analysis of high temperature response in *Pinus radiata*

Mónica Escandón [1□], Mónica Meijón [1], Luis Valledor [1], Jesús Pascual[2], Gloria Pinto[3], María Jesús Cañal [1]

[1]Plant Physiology, Department B.O.S., Faculty of Biology, University of Oviedo, Oviedo, Asturias, Spain.
[2]Molecular Plant Biology, Department of Biochemistry, University of Turku, Turku, Finland.
[3]Department of Biology and CESAM, University of Aveiro, Aveiro, Portugal.
[□] Current affiliation: Department of Biology and CESAM, University of Aveiro, Aveiro, Portugal.

***Correspondence:**
Corresponding Author: mjcanal@uniovi.es; escandonmonica@uniovi.es.

SUPPLEMENTARY TABLES AND FIGURES

1.1 Tables

Table S1: Identification of polar (methanol soluble) and nonpolar metabolites. A total of 2,317 peaks were analyzed after UPLC-Orbitrap (Table S1a) and CG MS (Table S1b) analyses. 40 peaks were unequivocally identified (those metabolites that were defined after the comparison to our compound library or by comparison of the MS/MS to online databases), and 776 peaks were tentatively assigned after comparing their accurate mass to that of reference compound databases. Peak information corresponding to UPLC-MS (1Sa) and GC-MS (1Sb) analyses is given.

Table S2: List of primers used for RT-qPCR including candidate genes (*PAL, DFR*, *DESATURASE* contig57599 and *DESATURASE* contig04128) and endogenous genes (*ACTIN, RIBOSOMAL PROTEIN 18S, GLYCERALDEHYDE 3-212 PHOSPHATE DEHYDROGENASE* and *UBIQUITINE*).

Table S3: Metabolomics pathways. **S3a)** Metabolites included in each KEGG metabolomics pathways; **S3b)** p-values metabolomics pathways obteined in MBROLE2 (http://csbg.cnb.csic.es/mbrole2/analysis.php).

Table S4: Matrices integrating dataset 1 for sPLS analysis. **S4a:** Polar and nonpolar metabolites. A total 2,317 ions were analyzed. ID, accession; Assigned/identified, compound name; m/z, neutral mass; RT, retention time (see Table S1 for detailed information about UPLC-MS dataset). **S4b:** Physiological parameters analyzed. Abbreviations: ABA, abscisic acid; SA, salicylic acid; ZR, zeatin riboside; GA7, gibberellin A7; iPA, isopentenyl adenosine; BK, Castasterone; IAA, indol-3-acetic acid; DHZR, dihydrozeatin riboside; iP, isopentenyl adenine; JA, jasmonic acid; GA9, gibberellin A9; MDA, malondialdehyde content; EL, electrolyte leakage; PSII, Effective photochemical quantum yield of PSII (φPSII); Proline, proline content; RWC, water content; Starch, starch content; TSS, total soluble sugars; fv.fm, maximum quantum efficiency of photosystem II; Chla, chlorophyll a content; Chlb, chlorophyll b content; Carot, carotenoid content.

Table S5: Number of common metabolites for all combinations of treatments and metabolites unique to those present in a single treatment.

Table S6: Partial Least Squares regression (sPLS): Metabolites and physiological parameters combined dataset with **(a)** X Loadings **(b)** Y loadings are shown.

Table S7: Principal components analysis (PCA) using metabolites and physiological parameters combined dataset. **(a)** Proportion of variance explained for each principal component (PC), **(b)** PCA loadings are shown.

1.2 Figures

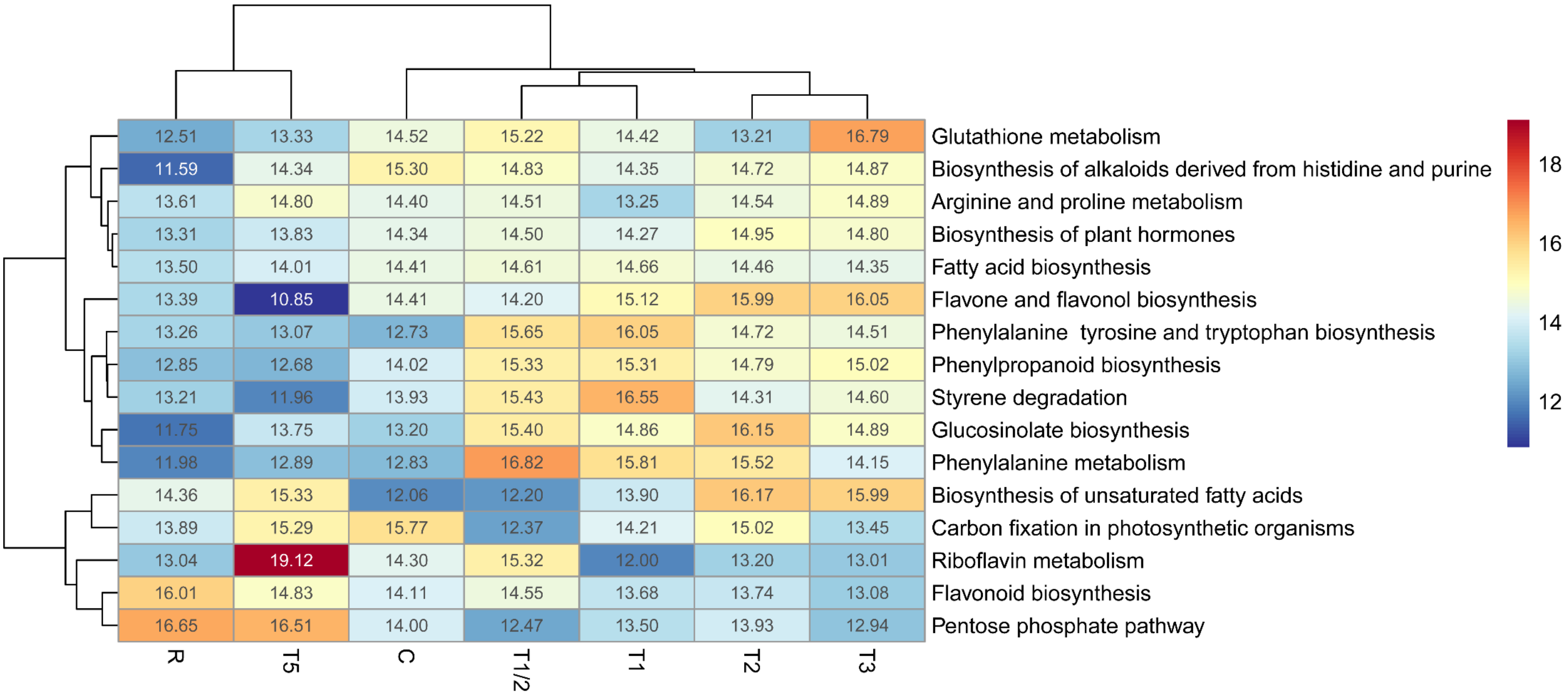

Figure S1. Heatmap-clustering analyses of main significantly altered KEGG pathways during high temperature response. Numbers inside the boxes indicate normalized abundance of each pathway (as a percentage) calculated as the sum of all identified/assigned metabolites within each pathway according to KEGG pathway. Abbreviations: control (C); recovered (R), 3 hours after 40 °C on day 1 (T1/2), 6-h heat exposure on day 1 (T1), day 2 (T2), day 3 (T3), and day 5 (T5).

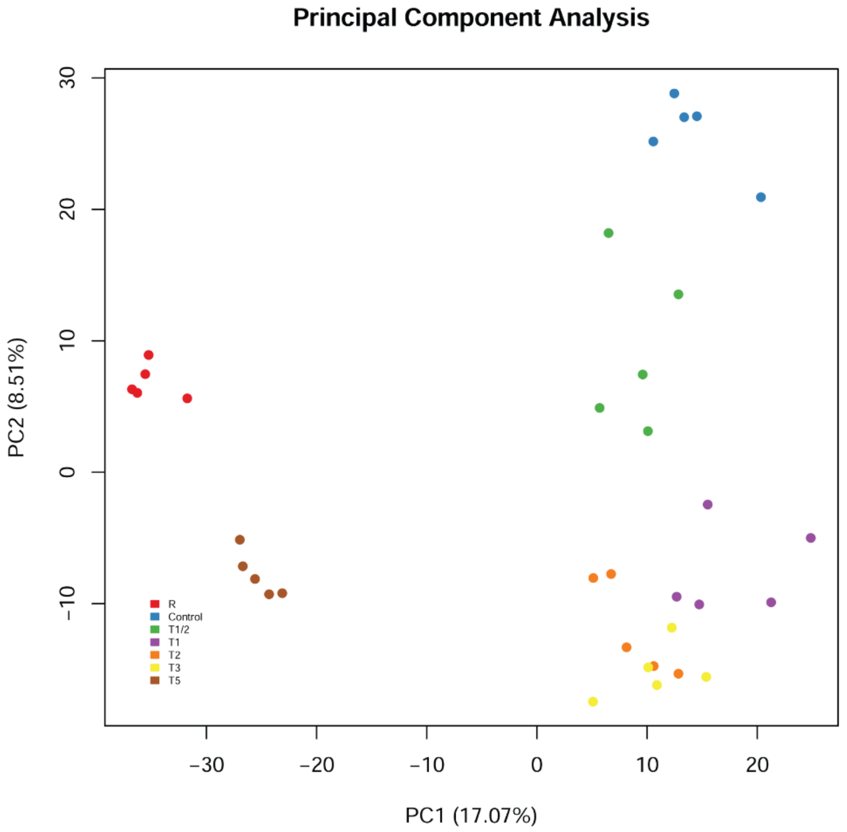

Figure S2. **Multivariate analysis of the metabolome, hormone and physiological parameters in needles during heat treatment and control plants (C, T1/2, T1, T2, T3, T5 and R).** Classification of the different samples according to Principal Components Analysis (PCA).

1.3 Video

Movie 1: Variations in the levels of hormones, metabolites, and physiological data during the analyzed time series. The sPLS network was built using metabolites and hormones as the predictor matrix and physiological parameters as the response matrix. (ID numbers represent the ID of metabolites shown in Supporting Information Table S1). Edge color represents the correlation value between hormones, metabolites and physiological parameters, while the size of the dot indicates the levels of each element at the different times. Only those correlations equal or higher, in absolute value, than 0.81 are shown. Color nodes reflects the "*amount of control*" that this node exerts over the interactions of other nodes in the network (higher control = lighter color). Abbreviations: electrolyte leakage (EL), zeatin riboside (ZR), isopentenyl adenosine (iPA), indol-3-acetic acid (IAA), dihydrozeatin riboside (DHZR), Castasterone (BK), gibberellin 7 (GA7), control (C); recovered plants (R), 3 hours after 40 °C on day 1 (T1/2), 6-h heat exposure on day 1 (T1), day 2 (T2), day 3 (T3), and day 5 (T5).

1.4 Data

Data S1: Interpretation of MS2 spectra corresponding to parent m/z 320.05, 739.16 and 519.11 tentatively identified as dihydromyricetin (n311), kandelin A-1 (n1059) and eujambolin (n780) respectively.

m/z 320.05 ($C_{15}H_{12}O_8$) tentatively identified as dihydromyricetin (n311) has main fragment ions at m/z 192.8 ($C_9H_6O_5$, loss of 1,2,3-benzenetriol, $C_6H_6O_3$) and m/z 300.9 ($C_{15}H_{11}O_6$, loss of one H_2O).

m/z 739.16 ($C_{39}H_{32}O_{15}$) tentatively identified as kandelin A-1 (n1059) has main fragment ions at m/z 453.0 ($C_{24}H_{22}O_9$, loss of luteolin, $C_{15}H_{10}O_6$), m/z 284.8 ($C_{15}H_{10}O_6$ it is loss one proton, $C_{15}H_9O_6$) and m/z 721.3 ($C_{39}H_{30}O_{14}$, loss of one H_2O).

m/z 519.11 ($C_{24}H_{24}O_{13}$) tentatively identified as eujambolin (n780) has main fragment ions at m/z 313.8 and 314.8 ($C_{16}H_{11}O_7$, loss of 2-[(3R,4R,6S)-3,4,5-trihydroxy-6-methyloxan-2-yl]acetate, $C_8H_{13}O_6$), m/z 204,8 ($C_8H_{13}O_6$, loss of one 3'-O-methyltricetin, $C_{16}H_{11}O_7$) and 503.8 ($C_{23}H_{21}O_{13}$, loss of CH_3).
