# Supplementary material for: Metabolome Integrated Analysis of High-Temperature Response in Pinus radiata
Source: Front Plant Sci. 2018 Apr 17;9:485. doi: 10.3389/fpls.2018.00485 (PMC5914196; doi:10.3389/fpls.2018.00485)
Supplement: Supplementary file 10 [file Data_Sheet_1.DOCX]

**Data S1:** Interpretation of MS2 spectra corresponding to parent m/z 320.05, 739.16 and 519.11 tentatively identified as dihydromyricetin (n311), kandelin A-1 (n1059) and eujambolin (n780) respectively.

m/z 320.05 (C_15_H_12_O_8_) tentatively identified as dihydromyricetin (n311) has main fragment ions at m/z 192.8 (C_9_H_6_O_5_, loss of 1,2,3-bencenetriol, C_6_H_6_O_3_) and m/z 300.9 (C_15_H_11_O_6_, loss of one H_2_O).

m/z 739.16 (C_39_H_32_O_15_) tentatively identified as kandelin A-1 (n1059) has main fragment ions at m/z 453.0 (C_24_H_22_O_9_, loss of luteolin, C_15_H_10_O_6_), m/z 284.8 (C_15_H_10_O_6_ it is loss one proton, C_15_H_9_O_6_) and m/z 721.3 (C_39_H_30_O_14_, loss of one H_2_O).

m/z 519.11 (C_24_H_24_O_13_) tentatively identified as eujambolin (n780) has main fragment ions at m/z 313.8 and 314.8 (C_16_H_11_O_7_, loss of 2-[(3R,4R,6S)-3,4,5-trihydroxy-6-methyloxan-2-yl]acetate, C_8_H_13_O_6_), m/z 204,8 (C_8_H_13_O_6_, loss of one 3'-O-methyltricetin, C_16_H_11_O_7_) and 503.8 (C_23_H_21_O_13_, loss of CH_3_).
