# Supplementary figures and images for: Metabolome Integrated Analysis of High-Temperature Response in Pinus radiata
Source: Front Plant Sci. 2018 Apr 17;9:485. doi: 10.3389/fpls.2018.00485 (PMC5914196; doi:10.3389/fpls.2018.00485)

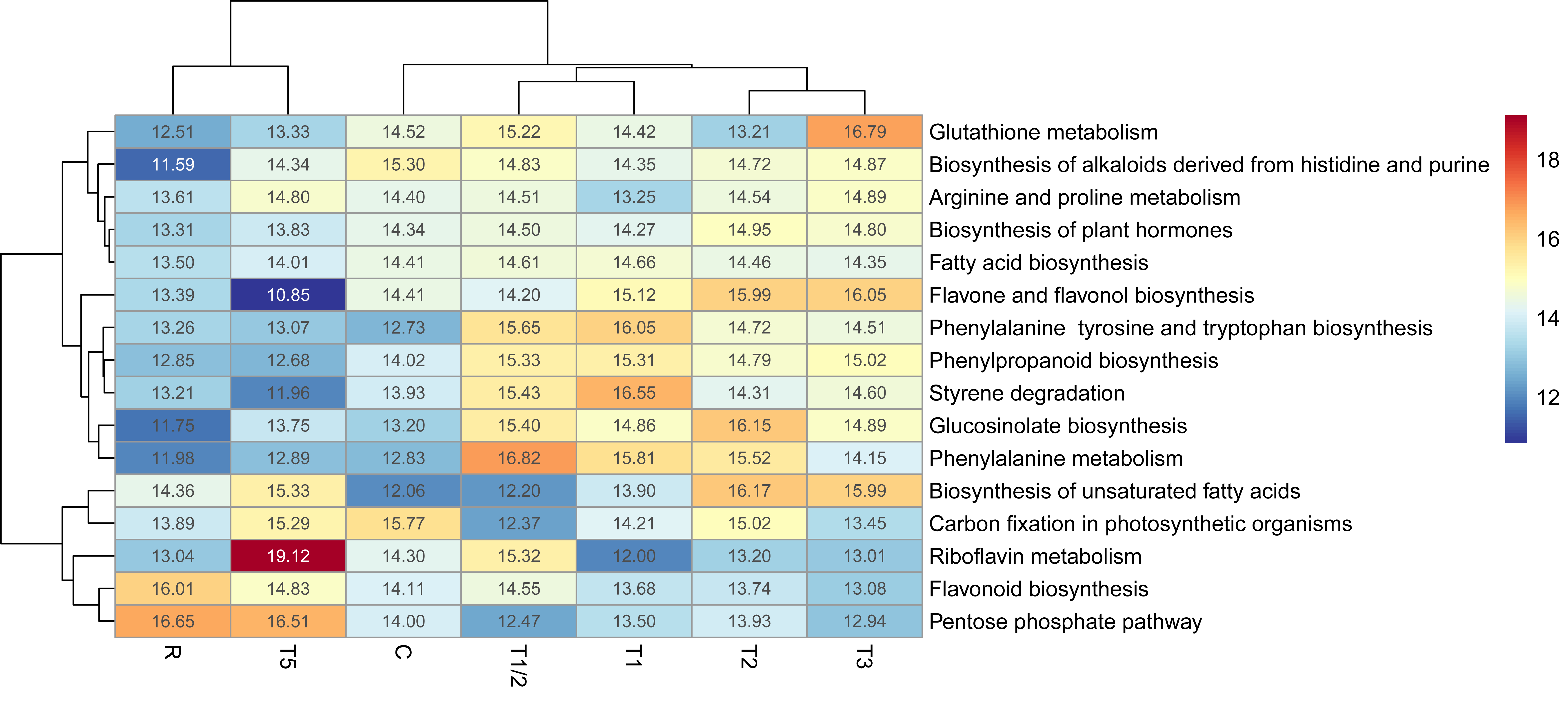

Supplement: Supplementary file 11 [file Image_1.TIF]

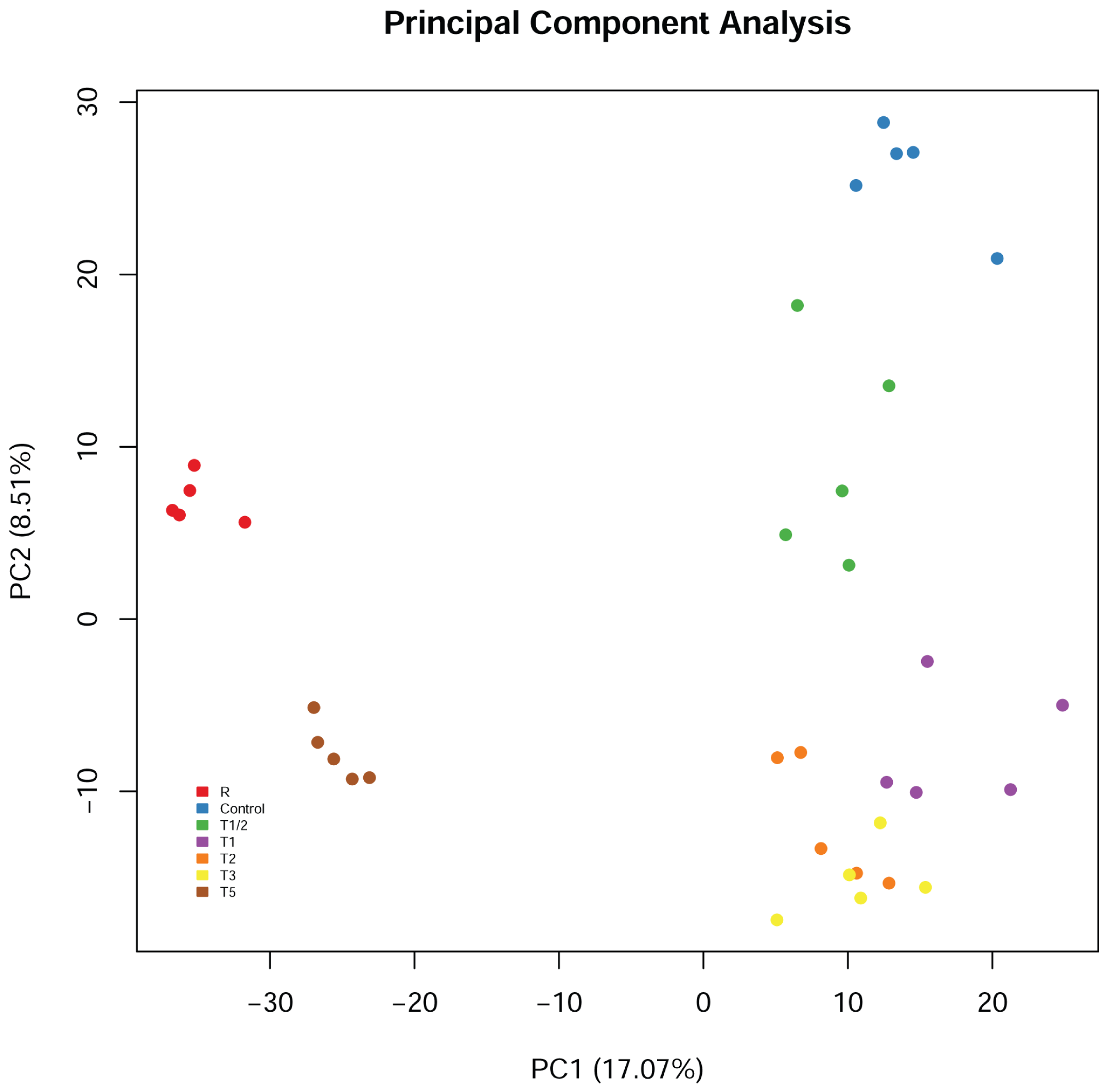

Supplement: Supplementary file 12 [file Image_2.TIF]
